# Stigma manifestations in cardiomyopathy care impact outcomes for black patients: a qualitative study

**DOI:** 10.1186/s12872-023-03556-6

**Published:** 2023-11-10

**Authors:** Morgan Wolfgang, Laura Beskow, Gillian Hooker, Mya Roberson, Katherine Anderson

**Affiliations:** 1 Vanderbilt University, Nashville, TN USA; 2 Vanderbilt University Medical Center, Nashville, TN USA

**Keywords:** Stigma, Cardiomyopathy, Qualitative, African-americans, Discrimination, Cardiology, Genetic, Racial disparity

## Abstract

**Introduction:**

Inequities in clinical care may contribute to racial disparities observed in studies of heart disease morbidity and cardiogenetic testing outcomes. There is a lack of research aimed at understanding the complexity of those inequities, but stigma likely contributes. This qualitative exploratory study helps close that gap in the literature by describing intersectional stigma manifestations perceived by the Black cardiomyopathy patient population at one academic medical center.

**Methods:**

Qualitative interviews were conducted with 14 Black cardiomyopathy patients. Interviews aimed to elicit patients’ experiences with discrimination related to diagnosis, symptoms, genetic testing, knowledge of genetic results, genetic counseling, providers’ actions, and providers’ communication. The interview guide was informed by The Health Stigma and Discrimination Framework. Data were also collected about participant demographics, type of cardiomyopathy, age of diagnosis, documentation of relevant family history, and completion of genetic counseling and/or genetic testing.

**Results:**

More than half of participants reported intersectional stigma manifestations related to their race, age, and/or weight while receiving care from cardiologists, nurse practitioners, genetic counselors, or clinical support staff. Stigma manifestations included physical roughness during patient care, withholding diagnostically-relevant information from the patient, impersonal care, coercion, and use of offensive stereotyped language by providers. These stigma manifestations impacted access to care, uptake of genetic testing, timeline to diagnosis, patient emotion, patient-provider trust, and adherence to medical recommendations.

**Conclusions:**

This study provides nuanced qualitative descriptions of stigma manifestations that affect patient and clinical outcomes in cardiology care and genetic services in one medical center in the Southeastern United States. The results of this study suggest that provider bias and stigma manifestations have an adverse effect on cardiogenetic and clinical outcomes among Black cardiomyopathy patients. Clinical interventions are suggested to assist health professionals in providing culturally-competent and respectful care. These results help inform patient-provider communication, clinical policies, and evidence-based practice in cardiology care and genetics. Continued study of this topic across more institutions and with a larger sample size is needed to confirm the generalizability of the conclusions.

**Supplementary Information:**

The online version contains supplementary material available at 10.1186/s12872-023-03556-6.

## Introduction

For many years, heart disease has been the number one cause of death in the United States [2]. Cardiomyopathy describes diseases, acquired or genetic, that affect the heart muscle. Symptoms include arrhythmia, chest pain, difficulty breathing, fainting, heart failure, and even sudden cardiac death [3]. Monitoring and treatment may include regular imaging (magnetic resonance imaging, echocardiography, etc.), trialing medications, and even heart transplant [3]. The most common type of cardiomyopathy is hypertrophic cardiomyopathy (HCM), which occurs in 1 out of every 500 people [3]. Some forms of cardiomyopathy result from alcohol and drug use, viral infection, or frequent intense exercise, but a significant proportion of cardiomyopathies have a genetic cause. A causative pathogenic variant may be identified in 60% of hypertrophic cardiomyopathy patients [1] and in up to 40% of dilated cardiomyopathy patients [14]. Genetic testing can be offered to cardiomyopathy patients to confirm a suspected clinical diagnosis and guide management [1]. Additionally, presymptomatic genetic testing can allow at-risk relatives to access medications that can significantly improve prognosis and lessen future symptoms [17].

Heart failure statistics, measured by the National Health and Nutrition Examination Survey, reveal striking racial disparities. The rate at which heart disease progressed to heart failure increased in Black patients by 67% from 1999 to 2016, while the progression to heart failure in white patients remained the same. After adjusting for age, sex, socioeconomic status, and cardiovascular risk factors like hypertension, obesity, or diabetes, patients were 1.5 times more likely to experience heart failure if they were Black [18]. This counters the assertion that Black people experience more heart failure because of lifestyle choices or fewer economic privileges.

Stigma is the internalized negative belief one holds about another person based on perceived characteristics [13]. Stigma related to race, gender, a health condition, and/or age may be experienced all at once (intersectional stigma) or separately [24]. Discrimination occurs when internal stigma manifests externally in one’s behaviors and attitudes, which may cause one to treat another person differently [13].

Providers’ internal stigma can lead to external discrimination, and discrimination impacts clinical and patient outcomes [21]. Oliver et al. found physicians reported preferences for white patients over Black patients. This study concluded that implicit and explicit racial stigma are significant in the physician population [16]. Stigma negatively impacts patients’ health behaviors, mental health, and physical health [24].

Furthermore, minority patients may be benefiting less from genetic services than white patients. About 1 in 28 people of African ancestry inherit a genetic predisposition to ATTR-CM [17]. Commonly, this is a specific founder mutation (p.Val142Ile) in the *TTR* gene [5]. However, minority populations are less likely to be offered clinical genetic testing by a physician or genetic counselor [4]. One study found that only 11% of patients with heart failure who have the Val142Ile mutation have an official diagnosis of ATTR-CM [6].

Despite known racial inequities, there is a lack of research aimed at understanding the complexity of how intersectional stigma is experienced by patients during cardiology care. There is also little research published about drivers and mechanisms of intersectional stigma [24]. Black Americans are disproportionately dying from heart failure, but most studies that report on the patient experience with heart failure or cardiomyopathy do not include minority perspectives. Many studies about patient experiences in cardiomyopathy care contain few or no black participants, and some studies fail to include race or ethnicity in the participant demographics at all [11, 19, 28, 29]. Thus, this study aimed to qualitatively explore Black cardiomyopathy patients’ experiences with intersectional stigma manifestations throughout their cardiology care and genetic services.

## Methods

We conducted qualitative interviews to explore Black cardiomyopathy patients’ experiences with intersectional stigma manifestations in cardiac-related healthcare and genetic services. The Vanderbilt University Medical Center Institutional Review Board deemed this research exempt under 45 CFR § 46.104(d)(2)(ii).

### Sample recruitment

Recruitment was limited to patients in the cardiovascular medicine division in one southeastern academic medical center in the United States between June 2020 and October 2021. Inclusion criteria included: having a diagnosis of cardiomyopathy, speaking English as the primary language, and self-identified race being Black or African-American in medical record intake forms. Patients who identified as multiple races/ethnicities were eligible if one of those races/ethnicities was Black or African-American. Patients were excluded if they were younger than 18 years of age or did not have access to a phone or internet services.

A total of 63 Black cardiomyopathy patients were invited to participate by secure messaging through a medical chart communication portal. Of the 63 patients invited, 15 participants consented. Fourteen qualitative interviews were conducted. One participant who consented did not respond to efforts to schedule the interview. After consent was received, retrospective medical chart reviews were completed to gather data about their demographics, type of cardiomyopathy, age of diagnosis, documentation of relevant family history, and completion of genetic counseling and/or genetic testing.

### Study design

The Grounded Theory Method informed this study’s design. It is the most commonly used method in qualitative research since it was introduced in 1967 by Glaser and Strauss. The Grounded Theory Method is an exploratory process in which portions of interview transcripts are simultaneously labeled, sorted, analyzed, and compared to earlier interviews. Data analysis occurs continually throughout data collection [12].

The interview questions aimed to elicit participants’ experiences related to diagnosis, symptoms, genetic testing, knowledge of genetic results, genetic counseling, providers’ actions, and providers’ communication. To avoid influencing participant responses, the interviewer did not mention race or discrimination until the last question of the interview. The interviewer asked probing questions about race or discrimination only after a participant raised the topic. The interview guide can be found in the supplemental documents.

### Data collection

The lead investigator (MW) conducted one-on-one interviews with the participants over the phone or via video call. Interviews were audio recorded with permission from the participants. Participants had no prior relationship or contact with the interviewer before recruitment. The fourteen semi-structured interviews had an average duration of 26 min (ranging from 12 to 56 min). The interview recordings were transcribed, and any participant or provider identifiers were removed from the transcripts before analysis.

**Fig. 1:**
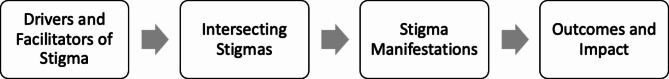
The relationship between intersectional stigma, stigma manifestations, and outcomes based on Stangl’s Health Stigma and Discrimination Framework [23]

### Framework

The interview guide was informed by The Health Stigma and Discrimination Framework. Stangl’s framework describes the process of intersectional stigma (its facilitators, manifestations, and the outcomes it can have on an individual and society) as depicted in Fig. 1 [21]. This framework has been applied to research and clinical practice for many health conditions, including non-communicable diseases [21]. This framework describes the intersectional stigma that can arise when stigma related to a health condition and stigma related to race, gender, or other characteristic are felt together by an individual. There are multiple factors that can drive intersectional stigma, which include stereotypes, cultural norms, lack of awareness, and prejudice. Stigma manifestations can impact outcomes, including access to care, uptake of testing, adherence to treatment, self-efficacy, social and legal protections, and quality of healthcare [21].

### Analysis

Data analysis was an iterative and continual process of collecting data, open coding, comparison, deductive reasoning, and thematic analysis. Data was managed and analyzed within MAXQDA software. Codes were generated inductively by reading and analyzing each transcribed interview. Themes were derived from but not limited to The Health Stigma and Discrimination Framework. One primary coder independently coded each interview transcript. A second coder analyzed 1 out of every 4 transcripts independently. The research team, which included individuals trained in qualitative analysis, reviewed and provided feedback on the emerging codes and themes within the transcripts. Coder 1 and Coder 2 met to cross-check and discuss codes, themes, interview experiences, and resolve analytic challenges before data analysis was complete. The researchers followed The COnsolidated criteria for REporting Qualitative research (COREQ) to ensure rigor and reliability [22]. The researchers were also guided by Williams and Morrow’s pan-paradigmatic perspective on achieving trustworthiness in qualitative research [27].

## Results

### Participants

Fourteen patients were interviewed for this study representing diverse perspectives (Table 1). Over half were women and nearly all self-identified as Black/African American. Participants were diagnosed with cardiomyopathy at an average age of 30.4 years (range: birth to age 55). Most had hypertrophic cardiomyopathy, although other types were also represented.

### Participants’ experiences

Eight of the fourteen participants reported experiencing stigma manifestations (discriminatory behaviors) during their cardiology care based on their attributes (race, weight, and/or age). Six of the fourteen participants reported experiencing stigma manifestations during their cardiology care specifically based on their race.

**Fig. 2:**
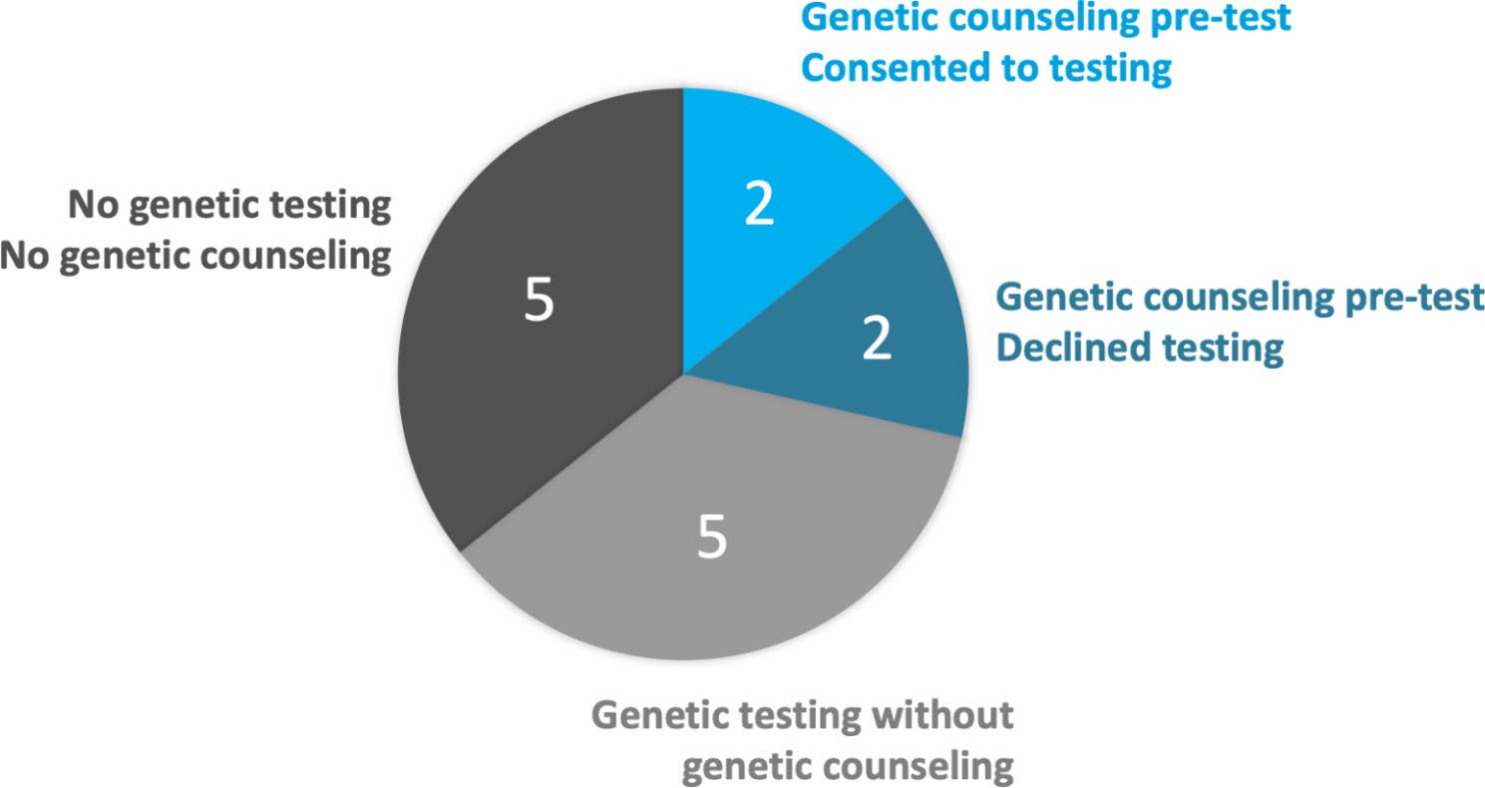
Genetic testing and/or genetic counseling following cardiomyopathy diagnosis

Seven participants had completed genetic testing, but only two of those seven had completed an appointment with a certified genetic counselor (Fig. 2). Two participants met with a certified genetic counselor but declined genetic testing.

**Fig. 3:**
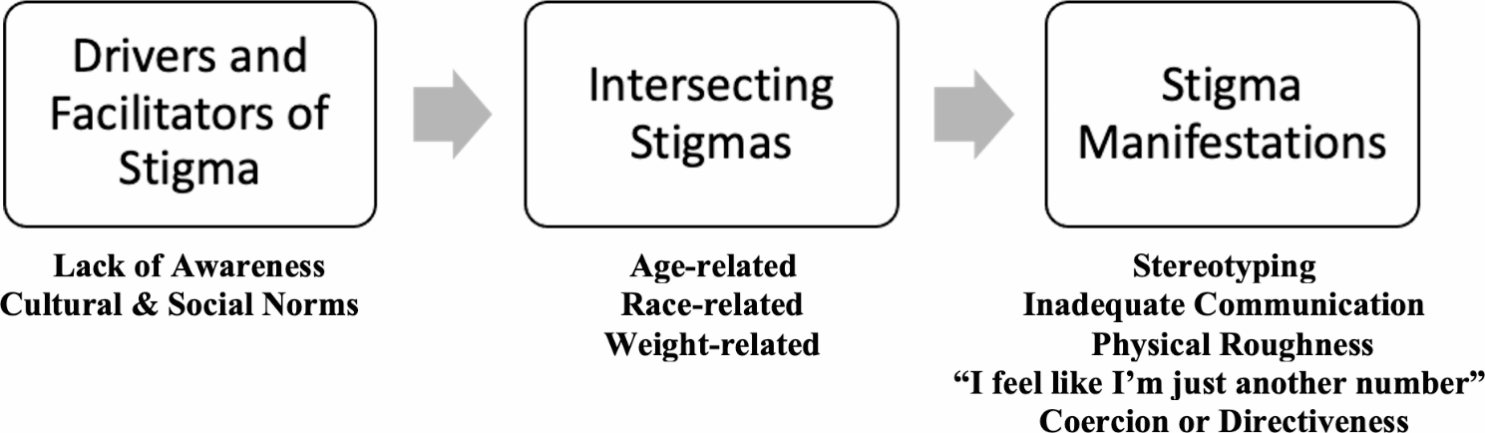
Themes and Subthemes

The participants described variable experiences with stigma and discrimination in cardiology care and genetic services. The data align with The Health Stigma and Discrimination Framework and were summarized into drivers and facilitators of stigma, intersectional stigma, and stigma manifestations (Fig. 3).

**Table 1:** Characteristics of Study Participants

Characteristics	n = 14	Number of Participants
***Gender***		
	Male*	6
	Female*	8
***Self-identified Race***		
	Black/African-American	13
	Black/African-American & White/Caucasian	1
***Age at diagnosis***		
	< 1 year (Neonatal period)	2
	1–9 years	0
	10–19 years	1
	20–29 years	3
	30–39 years	4
	40–49 years	2
	50–59 years	2
***Age at time of interview***		
	20–29 years	1
	30–39 years	4
	40–49 years	5
	50–59 years	3
	60–69 years	1
***Type of Cardiomyopathy***		
	Hypertrophic Cardiomyopathy	8
	Dilated Cardiomyopathy	4
	Transthyretin Amyloid Cardiomyopathy	1
	Unspecified in chart**	1
***Family history of SCD and/or heart disease***		
	Yes	10
	No	4

*Assigned this sex at birth

** Genetic testing identified a pathogenic variant in the *TTR* gene, and this participant has symptoms that are associated with ATTR-CM. However, no amyloidosis evaluation had been completed at the time of data collection, and no diagnosis was assigned

### Drivers and facilitators of stigma

Interviews revealed providers’ lack of awareness as a factor that enabled stigma to develop (driver) and cultural norms as a factor that contributed to continuation of stigma (facilitator).

1. Providers’ Lack of Awareness

One participant recounted how lack of awareness of dermatologic findings on darker skin impacted her health.

*I remember how my mother used to fuss at me. She would tell me that I was not bathing correctly, that I was not washing my neck, and I was washing my neck. It’s just that my neck has always been dark. My inner thighs have always been dark, and my underarms have always been darker than the rest of my body. And that’s why the doctor at the diabetes clinic said that I have actually always been diabetic… and he is the first and only person that has ever said that to me. And, it very well could have been one reason for the heart failure, that I possibly lived with diabetes uncontrolled all this time.*

Her past providers’ lack of awareness of dermatologic findings on darker skin may have been a driver of stigma. She described signs of acanthosis nigricans in childhood, which is indicative of diabetes, and reported that no providers had previously mentioned diabetes until she was already diagnosed with cardiomyopathy at the age of 36. The lack of awareness of Black dermatologic findings may have resulted in lack of proper workup for diabetes at a younger age, which delayed diagnosis, decreased her access to diabetic care, and resulted in worse cardiac outcomes.

2. Cultural Norms

Another participant described how her upbringing, specifically that she was raised to not voice complaints, resulted in her silence during a physically painful medical encounter.

*I just felt like, “Why are you so uncomfortable with me? I’m sitting here, and I really can’t do anything. I can barely move. Why would you not be gentle with me?” But I would never say that because I don’t feel like I can.… The way I was raised is… if someone is not great to you, we don’t talk about it or you kind of suppress it. You push it down… I just didn’t even acknowledge them just because that’s the way I’m programmed in my head… It’s just unfortunate… You can’t help but feel like it’s attributed to your race.*

This participant’s experience exemplifies how her culture and social norms influenced her reaction to stigma. Her upbringing made it feel inappropriate to comment on or report the stigma manifestation (physical roughness). This account highlights that there is a known power dynamic between clinicians and their patients that may exacerbate patients’ ingrained cultural norms and facilitate stigma.

### Stigma manifestations

The participants described a wide-range of experiences with stigma manifestations during their care.

1. Stereotyping

Several participants spoke of negative encounters with providers and support staff that centered around inaccurate stereotypes. These participants reported stereotyping that has historically been experienced by Black people more than other races, particularly stereotypes related to sexual promiscuity, substance abuse, and inability to pay for services.

One participant experienced providers’ assumptions of risky sexual behaviors and promiscuity, which she perceived as offensive stereotyping.

*I thought I was pregnant. [The cardiologist] told me that I need to be using condoms, like Black people just go around here not using protection… And I do use [condoms], even if I have been dealing with the same person for 10 years. Black people don’t just go around here having sex all day and all night and whatever the case may be unprotected… And it made me view her and look at her in a whole different light… That offended me and affected me so bad to where I was embarrassed, and I’m even embarrassed now… Can I trust to go to a clinic?… Because I already have trust issues with people just in general. Now I’m getting to where I can’t even trust to go to the doctor… [Providers] say certain things to people of color that they probably wouldn’t say to someone like you [white interviewer].*

The participant also shared that the provider did not ask about her birth contraceptive methods before progressing to education about condom use. The participant reported she had no opportunity to further explain before being lectured about unprotected sex. This stereotyping led to the participant’s embarrassment and distrust in the provider. This made the participant question whether it was worth it to go to future appointments and regular check ups. This stereotyping also resulted in the participant anticipating future discrimination, which led to hesitation about future health communication.

Another participant reported an experience when he presented to the emergency room.

*They were looking at me like, “Okay, he’s just trying to get drugs”… Someone that’s constantly sick and that’s just skinny for no apparent reason, automatically you go to assume, “Hey, he’s probably a crackhead” without really actually knowing the person you’re dealing with… You get judged on what they see before.*

This encounter exemplifies intersectional stigma because racial- and weight-related stigmas were felt at the same time by this patient. The providers’ stigma manifested as stereotyped thinking that he was drug-seeking and disbelief that he was experiencing real symptoms. There was a reciprocal lack of trust between the patient and provider. These stigma manifestations affected clinical outcomes by inhibiting his access to quality care at the ER and delaying diagnostic testing and medical intervention.

Another participant also experienced stereotyping in her care. She described a telephone encounter with scheduling staff who made assumptions, which limited her access to care.

*…I assume that they think that I may be from a different culture because my name is not typical. I was a little bit upset because I had agreed with my cardiologist that for cardiology care I’d go every six months… I was reviewing my list that was given to me of when the next appointments are. I called [the scheduler], and I was like, “This is not correct because I’m supposed to go every six months, not every year to the cardiologist…” Then the scheduler told me, “I’m not sure if you can afford this. You already have a lot of other appointments with our other care teams. So this is what we want to give you.“ And I’m thinking to myself, “How can you jump to so many conclusions?”… That was really off putting for me… Why am I fighting for care?*

This participant perceived that the scheduler made assumptions based on the demographic information listed in the chart. The stereotyped stigma manifestation was that the scheduler assumed she was unable to afford cardiology appointments every six months. This inhibited her ability to adhere to her cardiologist’s medical recommendations.

Another participant described a negative experience during family history discussion.

I don’t even know if I want to go back to see that genetics counselor.… She’s like asking me how many kids my mom had. And I said three. And then she asked me, “Well, do all of y’all have the same dad or different dads?” Black people do not have all different baby daddies or whatever…we all have the same dad, all three of us…I know they have to know all this stuff for genetic reasons, but I just feel like the questions and the way they were asking was very inappropriate or offensive stuff. I felt like even if you feel a certain kind of way, try to clean it up a little bit, don’t make it seem so obvious that’s how you feel.

She perceived that the provider assumed her mother may have conceived children with multiple men, which is historically a stereotype associated with Black families. This led to hesitation towards returning to the genetic counseling clinic in the future; this stereotyped interaction is a barrier to her future care.

2. Inadequate Patient-Provider Communication

Multiple participants voiced concerns about inadequate provider communication and providers withholding diagnostically-relevant information.

One participant reported a provider withheld important information about her positive genetic results that revealed a pathogenic variant in the *TTR* gene.

*[The cardiologist] did the genetic saliva test, and she told me this was harming. [The cardiologist] was like, “You have a disease that old people get.“ Well, I didn’t know what that meant. So [the cardiologist] was like, “Well, we won’t discuss that until you start having the symptoms.” She never told me the name of it. I started having this tingling in my hands and feet. That’s when I told her. [The cardiologist] had me come in, and then she told me about TTR symptoms.*

This provider withheld information about her genetic results for 11 months. Age-related stigma manifested as poor provider communication and education about the genetic test result. This inhibited the patient’s ability to understand the implications of her genetic results, adapt to potential risks, and make informed decisions about her healthcare and family planning. She was also unable to share the genetic results with at-risk relatives. Additionally, in the absence of an early discussion about ATTR-CM, the patient couldn’t know that the tingling she felt was related to TTR cardiomyopathy. The lack of communication about genetic results meant this participant was at risk for delayed symptom identification, which could delay access to targeted medications and impact health outcomes.

One participant detailed how inadequate provider communication can foster distrust.

*[The cardiologist] didn’t do a good job explaining the symptoms. I think they didn’t do a good job explaining treatment… I just don’t think that doctor did a good job explaining the seriousness of it… I just don’t trust doctors there… I didn’t think that they were concerned about my overall health… I probably say [racial stigma] has a little play into it. I couldn’t say to what degree. But I would probably say [racial stigma] contributed.*

This participant stated the provider didn’t adequately explain the potential morbidity of the condition and treatments he was undergoing. This led the participant to lose trust in the provider and question if the provider genuinely cared about his health. He states that he perceives this as a racial stigma manifestation.

Another participant did not recall that genetic testing had ever been ordered. When asked what he believed caused his heart problem, he voiced his confusion.

*Well, at one point I thought [the cardiologist] may have told me it could have been hereditary. Then they came back and said, “No, it’s not necessarily hereditary.“ So I really don’t know… [Silence] What do you mean genetic test?… I’m assuming that when you say genetic, are you saying in reference to other family members or just checking my blood? Its breakdown?*

A chart review revealed he had a pathogenic variant in the *TTR* gene that contributed to his diagnosis of ATTR-CM, and he had never been referred for genetic counseling. A referral to a certified genetic counselor could have helped this patient cope with the diagnosis and improve understanding of his genetic results. Of note, this participant has living children and siblings, who would each have a 50% chance of inheriting the causative genetic variant. It is unclear if the relatives have been informed of their genetic risk.

3. Physical Roughness

A few participants shared their experiences with providers who were not gentle during care. These participants perceived providers’ racial stigma as the motivation behind the painful encounters.

One participant described her experience in recovery after her implantable cardioverter defibrillator (ICD) surgery when a provider was physically rough during their care.

*The person basically ripped the bandaid off of my skin, where my new suture was. It left a scar… I was in so much pain when the person did that. I was honestly afraid that it would detach my sutures with how they just ripped it off… and I have the scars. I keep getting frustrated because I shouldn’t have that scar… The care teams I see on a regular basis, I love them… It’s just most one-offs that happen… You can’t help but feel like it’s attributed to your race.*

This participant perceived the provider’s internal racial stigma led to the external stigma manifestation of physical roughness during this interaction.

Another participant described his nurses having difficulty locating a vein during a blood draw.

*They just kept sticking and sticking. It was a bit uncomfortable for me. I mean, I realized they had to do it, but it’s just a bit uncomfortable for me… Maybe they felt like I should be a little bit tougher due to my race, with the needle poking, because that’s been something that’s floated out there, that African-Americans can tolerate and have a higher threshold for pain. So that could have been some reasoning behind why they were doing what they were doing*.

His perception that nurses may perceive Black people as having a higher pain tolerance is backed by research. Recent reports found that medical personnel believe Black people feel less pain than white people [8].

4. “I feel like I’m just another number”

Some participants conveyed that they felt provider stigma contributed to impersonal and rushed appointments.

One participant describes his perception of how his race has played a role in his quality of care, specifically during urgent care or emergency room visits.

*You feel like they in a hurry to get rid of you, so they can get to somebody that they care more about. I know that doesn’t make sense, but it’s that way sometimes.*

This participant has experienced feeling like he wasn’t given adequate time and attention because of providers’ racial stigma.

Another participant describes how this feeling during appointments affects her ability to stay hopeful throughout her long-term cardiology treatment plans.

*There are times when I feel like I’m just another number. I understand I am not the only person with heart failure, but whenever you’re in front of your doctor, you want to feel like right now I’m the most important person, and let’s try to figure it out together… Sometimes it’s depressing. Sometimes I feel like giving up… I felt like I was going to die, that it was over for me, that [the providers] weren’t even trying anymore… And so when [the nurse practitioner] said to me, “We’ve done all we can do"… I don’t think she would have said that to someone else in the white race.*

She perceived the nurse practitioner’s internal racial stigma led to impersonalized care and lack of concern. Of note, after this participant transferred care to a new provider, who she perceived is less discriminatory, her feelings of hopelessness subsided. This participant reported she is now satisfied with her new care team, and she believes her current provider cares about her well-being and quality of life.

5. Coercion, Provider Too Directive

Some participants described ways in which they felt their cardiology team pressured them to complete research or interventions that they did not wish to pursue.

One participant described a provider who tried to enroll him in research during his care, even after he had declined the offer at previous cardiology visits.

*[The cardiologist] wanted me to participate in a study, but I refused that… I didn’t want to be a Guinea pig… I think [the cardiologist’s] intentions are well, but she likes to try to put you into a study… she tries to push a lot of those research… I know of history where African-Americans have been experimented on, but that wasn’t a factor in my thinking. I just didn’t see the value in [the study].*

Another participant described her perception that a provider was coercive in counseling about the benefits and risks of ICD surgery.

*I’m glad I didn’t do [the ICD surgery] because I’m getting treated with medicine. [The doctor] was leaning more to do that surgery within the next couple of months, and I was like, no… I never got the procedure done. It was like, give me a chance. [The same doctor] said, “The medicine can treat you.“ I think the medicine is doing very well for me.*

This patient felt pressured by the cardiologist to undergo ICD surgery in the near future, but the patient wanted to continue using medication to slow the progression of ATTR-CM. Explicit stigma wasn’t described within this particular interaction, but the conversation emphasized a lack of shared decision making.

**Table 2:** Impact of Intersectional Stigma Manifestations

Outcome	Number of Participants
Decreased access to care and services	5
Lack of patient-provider trust	4
Delayed diagnosis	3
Decreased adherence to recommendations	3
Patient frustration	3
Patient depression, hopelessness	2
Patient humiliation	2

### Impact of stigma manifestations

As these examples illustrate, stigma manifestations had a potentially significant impact on clinical and patient outcomes (Table 2). More than one third of the participants experienced decreased access to care and services as a result of stigma manifestations in the clinic. Four participants discussed a lack of trust in their relationship with the providers. Two participants specifically reported their distrust led them to question or reject medical recommendations made by that provider. Another participant intended to follow medical recommendations, but stigma manifestations created barriers to care. About one fifth of participants reported provider stigma that delayed diagnosis.

Stigma manifestations when interacting with providers and support staff also impacted participants’ emotions. Four participants shared that their emotions were negatively impacted by stigma manifestations, and they felt depression, hopelessness, frustration, and/or humiliation. Two participants reported that frustration and humiliation coincided during experiences with stigma.

Four participants experienced stigma manifestations that negatively impacted three or more outcomes. Of note, one participant experienced six out of the seven negative outcomes reported. This participant reported experiencing significant discrimination in multiple interactions with providers and support staff related to her race, which decreased her trust in providers, decreased her adherence to medical recommendations, and decreased her access to care and services. She stated these interactions with providers contributed to frustration, hopelessness, and humiliation.

## Discussion

This study aimed to explore Black cardiomyopathy patients’ experiences with intersectional stigma manifestations throughout cardiac-related healthcare and genetic services. The goal was to help close the gap in the literature by identifying some drivers of stigma and describing how stigma manifestations are perceived by the minority patient population.

The participants’ reports aligned with the process illustrated by Stangl’s Health Stigma and Discrimination Framework (Fig. 1). Participants reported that provider stigma was perpetuated by patient cultural norms. Participants also reported that providers’ stigma (particularly regarding the participants’ age, weight, and skin color) manifested as stereotyping, inadequate communication, physical roughness, impersonal care, and coercion.

There was high variability between participants’ reports of intersectional stigma. Every participant had distinct experiences with providers and unique interpretations of the stigma manifestations that occurred, which highlights the need for individualized, compassionate care and provider empathy in every appointment. Over half of the participants in this study reported experiencing discrimination in cardiology care and/or genetic services. These findings parallel statistics published in a recent quantitative study with a larger sample size that estimated 54% of Black patients in America experienced intersectional discrimination in healthcare [15].

The participants in this study reported that stigma manifestations impacted clinical and patient outcomes (Table 2). Some participants were fearful of future medical appointments due to anticipated stigma based on past interactions with providers. Some participants’ access to care and services were limited by the medical staff’s internalized stigma that manifested externally as discriminatory behaviors, such as disbelief of symptoms and perceptions of inability to pay for services. Inadequate communication between the provider and patient was reported in multiple interviews, and these reports correlate with previous publications. A systematic review of observational and patient-reported data concluded that Black patients received lower quality patient-physician communication than other races. The systematic review also showed that physicians share less information with Black patients compared to patients of other races [20].

Participants in this study also described their distrust in cardiology providers for a variety of reasons, which sometimes led to patients being unwilling to follow providers’ medical recommendations or return for follow-up. Many participants described emotional outcomes and dissatisfaction after facing stigma manifestations, particularly depression, frustration, and humiliation. The data align with past research that asserts stigma affects outcomes. Over a decade ago, Greer described racial stigma manifestations in cardiovascular healthcare that led to patients’ medical distrust, lower appointment attendance, and nonadherence to medical recommendations [7]. Our data align with Greer’s findings and support the assertion that these processes are still occurring today. Additionally, our study adds to Greer’s findings by contributing data about intersectional stigma manifestations and stigma manifestations related to genetic services.

Some participants also reported provider coercion or pressure to consent to research or elective surgical procedures. A systematic review of observational studies showed black patients spoke and engaged with providers less during appointments compared to white patients. Black patients also had less participation in decision-making than white patients during appointments [20]. Our data and the systematic review suggest that a patient’s lack of engagement in an appointment may be driven by engrained familial, cultural, or societal norms rather than lack of interest in shared decision-making. Therefore, providers should encourage Black patients to speak freely and ask questions. A conscious effort should be made to allow Black patients to make autonomous decisions free from coercion when planning healthcare management and when consenting for research.

Participants, both those who experienced discrimination and those who did not, voiced awareness of past racial injustices in healthcare. During interviews, several participants mentioned historical differences in care for Black patients compared to white patients, such as unethical research practices, increased physical roughness during care, and discriminatory access to health care. Previous studies have also shown that Black patients are aware of generalized physician bias and racial health disparities in America [10, 25]. To reduce these biases, all staff could benefit from education about the commonly-held false beliefs about Black patients’ pain tolerance and other biological falsehoods.

This study also highlights the value and importance of genetic counseling for cardiomyopathy patients and their families. The majority of participants who received genetic testing did not meet with a certified genetic counselor despite access to one at their institution (Fig. 2). The American Heart Association, The American College of Cardiology, The European Heart Rhythm Association, and The Heart Failure Society of America support the inclusion of genetic counseling in cardiomyopathy care and recognize the positive impact [23]. If genetic counseling is not accessible for a patient, an appointment with the ordering provider should be scheduled to discuss the genetic results. This dedicated time allows the patient to better comprehend implications and discuss strategies for informing at-risk relatives. To improve patient retention of information, providers can employ the teach-back method. The teach-back method asks the patient to repeat back a brief summary of results and share any takeaways they have, which helps providers identify gaps in understanding [26].

It is common practice to inquire about and document a patient’s ancestry or ethnicity and any consanguinity within the family [9]. Typically providers ask these questions while gathering family history information or during genetic risk assessment to predict which genetic conditions or inheritance patterns are more likely. However, genetic test options and medical care are typically unchanged regardless of a cardiomyopathy patient’s answers to these questions. Therefore, physicians and genetic counselors should consider if it is clinically relevant to ask about a patient’s ancestry or consanguinity. This information can be gathered if it will influence which genetic test is ordered or other medical management. If it is necessary to gather this information, providers should consider providing an explanation of why these questions are necessary before asking. Offering an explanation for probing questions can also be useful before assessing genetic relationships (e.g., half versus full siblings) or evaluating clinical risk factors. If the patient’s ancestry or consanguinity won’t change medical care, providers should not ask these questions.

Although not a primary focus of this study, some participants reported they had experienced health-related stigma and intersectional stigma in other settings, such as romantic relationships, friendships, family gatherings, airport security, police encounters, real estate purchases, and employment. This raises the question of whether experiences in society influence perceptions and outcomes of clinical interactions, but further research is needed to clarify this interplay between environments.

### Strengths and limitations

This exploratory study provides nuanced qualitative insight into the intersectional stigma experienced by Black patients during cardiomyopathy care at one major academic medical center. The sample includes participants with a range of ages and equal representation from both male and female genders to elicit any age-based or gender-based stigma. To avoid influencing participant responses, the interviewer did not mention race or discrimination until the last question of the interview. The interviewer asked probing questions about race or discrimination only after a participant raised the topic. The interviewer frequently summarized participant stories and emotions during interviews, so the participants were able to confirm or correct the interviewer’s understanding. However, participants were recruited exclusively from one academic medical center in the southeastern United States and cannot represent the full breadth of healthcare experiences among Black cardiomyopathy patients. Furthermore, many of the participants emphasized that their negative experiences did not take place at their current medical institution, and it was not always clear when or where the events occurred. Additionally, the inclusion/exclusion criteria limited the sample population to patients who were English-speaking and had access to phone or internet services, further limiting generalizability. There is also bias because a white researcher conducted interviews and analyzed transcripts of Black participants. To acknowledge and reduce this bias, the interviewer met with the research team after some of the interviews to discuss any shock, defensiveness, and general impressions. The interviewer also journaled about any countertransference or other reactions to identify any unconscious bias. This study does not include the healthcare providers’ perceptions of the interactions shared by participants. This study does not speculate whether these stigma manifestations arise from conscious or unconscious biases of medical staff.

Future directions for research include multi-institutional collaboration with a greater number of participants. More research is needed to determine if these results are generalizable to a broader population. Additionally, study of providers’ motivations and experiences with health equity in cardiomyopathy care would improve our understanding of this space.

## Conclusion

Black cardiomyopathy patients in this study described the intersectional stigma manifestations experienced in their cardiology care. Provider bias and stigma manifested most overtly as stereotyping, inadequate communication, physical roughness, impersonal care, and coercion. These stigma manifestations negatively impacted patient and clinical outcomes. This study suggests clinical improvements that can assist health professionals in providing culturally-competent and respectful care for Black cardiomyopathy patients. This data can inform patient-provider communication, clinical policies, and evidence-based practices in cardiology care and genetics.

### Implications for clinical practice

- It may be beneficial for cardiologists to place a referral to genetic counseling before genetic testing is ordered for Black cardiomyopathy patients. This ensures the patient is informed and has considered benefits and limitations before electing genetic testing. A genetic counselor would also disclose results in a thorough and timely manner and can streamline cascade testing for relatives. The United States directory is available at www.FindAGeneticCounselor.com. A similar directory for other countries may be useful.
- If genetic counseling is not accessible, an appointment with the ordering provider should be scheduled to discuss the genetic results. This dedicated time allows the patient to comprehend implications and discuss strategies for informing at-risk relatives. Providers can use the teachback method to ensure the patient understands results before leaving the appointment.
- Black cardiomyopathy patients experienced discrimination from other healthcare staff in addition to providers. Training about compassionate care, stereotyping, and microaggressions should be completed by all employees who interact with patients and patient data.
- Genetic counselors and other providers should consider if it is clinically-relevant to ask any patient, regardless of race, about their ancestry and possible consanguinity. This information can be gathered if it will change the test ordered or medical management. Consider providing an explanation of why these questions are necessary before asking.
- Some participants reported depression and hopelessness. Regular mental health and psychosocial assessment is warranted for this population. Resources and counseling can be provided depending on patient needs.
- Healthcare facilities should integrate tenets of health equity and diversity into their institutional policies. Policies can include guidance for training, expectations for service, and potential disciplinary measures.

### Electronic supplementary material

Below is the link to the electronic supplementary material.

Supplementary Material 1

## Data Availability

The datasets used and/or analyzed during the current study are available from the corresponding author upon reasonable request.

## Abbreviations

ATTR-CM: Transthyretin Amyloid Cardiomyopathy
DCM: Dilated cardiomyopathy
HCM: Hypertrophic cardiomyopathy
ICD: Implantable cardioverter defibrillator
SCD: Sudden cardiac death
