# Supplementary material for: Stigma manifestations in cardiomyopathy care impact outcomes for black patients: a qualitative study
Source: BMC Cardiovasc Disord. 2023 Nov 10;23:553. doi: 10.1186/s12872-023-03556-6 (PMC10638684; doi:10.1186/s12872-023-03556-6)
Supplement: Supplementary file 1 — Supplementary Material 1 [file 12872_2023_3556_MOESM1_ESM.docx]

**Supplemental Documents**

1. Interview Guide
2. EMR Chart Review Form
3. The COnsolidated criteria for REporting Qualitative research (COREQ)

**Semi-structured Interview Guide**

*Main questions are numbered. Probing questions are bulleted.*

**“This interview is completely separate from your healthcare. Your answers will not be a part of your medical record, and providers will not see your answers. Please feel comfortable sharing any positive or negative experiences. Your identity will not be connected to the responses.”**

1. Can you tell me what your official diagnosis is?

- Can you tell me what you understand about what is happening to your heart and body?
- Tell me about your journey to getting a diagnosis.
- How well has your cardiology care team explained your diagnosis to you?
  - On a scale from 1 to 5, 1 being they did a bad job and 5 being they did a really great job?
  - Tell me more about why you picked that number.

1. What do you believe caused the heart problems?

- Why do you believe this caused it?
- What do you think your doctor believes caused your heart problems?
- Did your provider ask about your family history?
- Do you feel you got a thorough workup?

1. Has a provider ever talked to you about the genetics or inheritance of this condition?

- If yes- Tell me about that conversation? Was it helpful? How did it affect your view of this condition?
- If no- Have you ever wondered if this is something that could run in your family?
- Are you interested in learning more about genetics?
- Do you have any distrust or hesitation surrounding a genetic consult? Why?

1. Sometimes people feel like others blame or judge them because of their health problems. I wonder if that is something you’ve experienced with your heart condition?

- Blame or judgment from yourself
- Blame or judgment from friends or family
- Blame or judgment from healthcare workers or doctors

1. Have you ever felt your race or ethnicity played a role in your care or affected interactions with the providers?

- Tell me about the one that stands out most in your mind.
- Tell me about the most recent experience.

**Medical Record Data Collection Form**

**
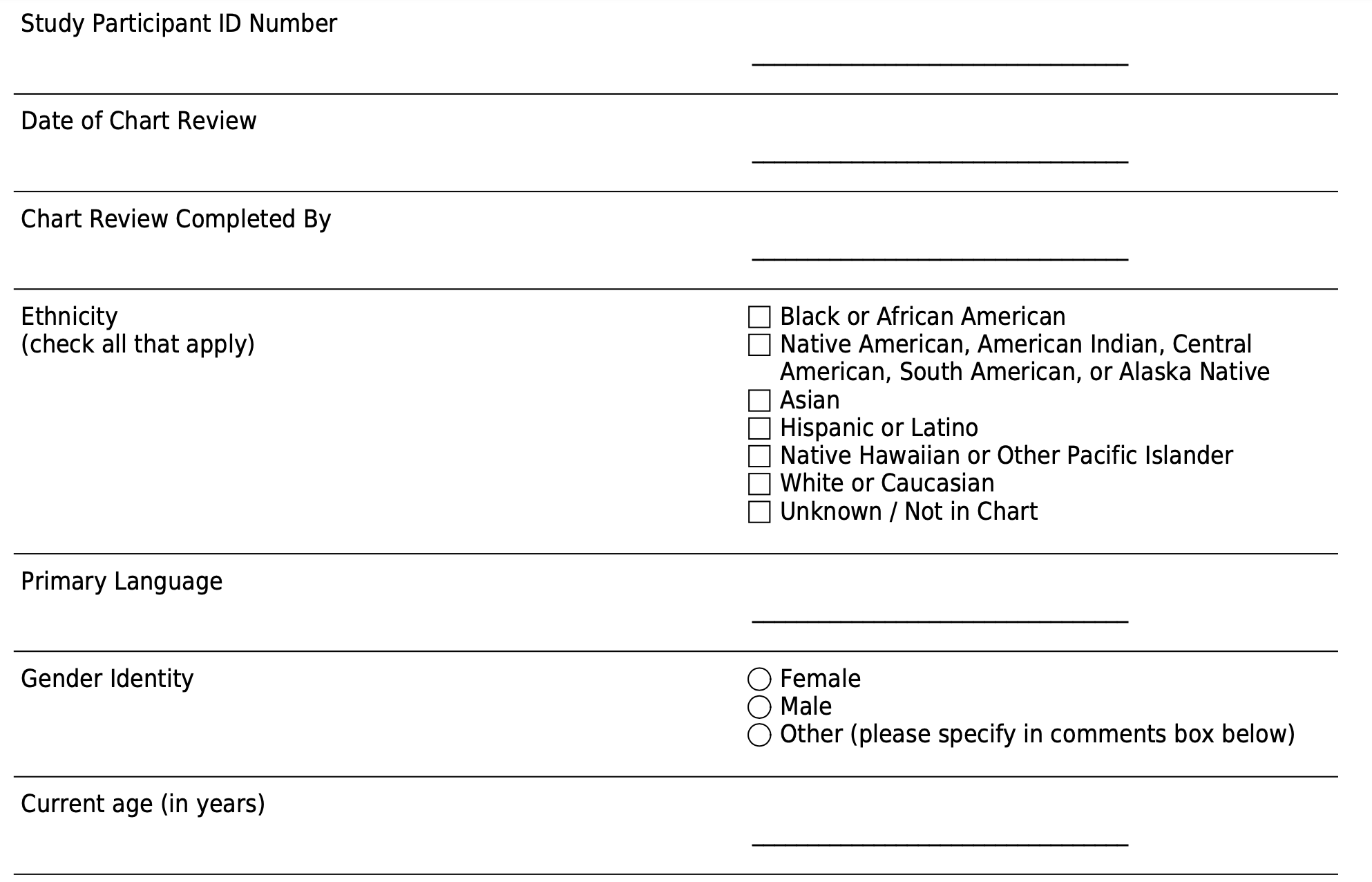
**


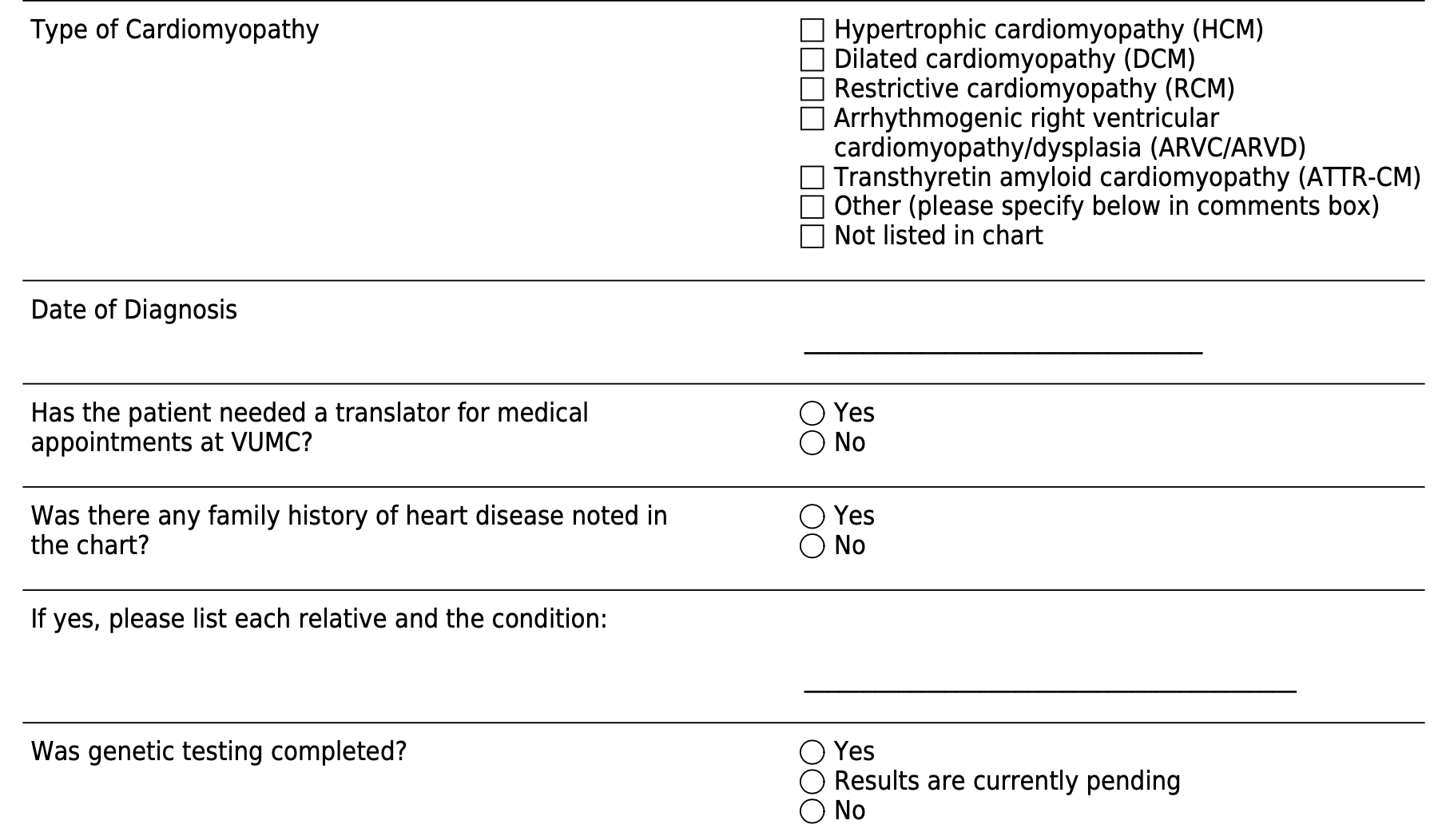


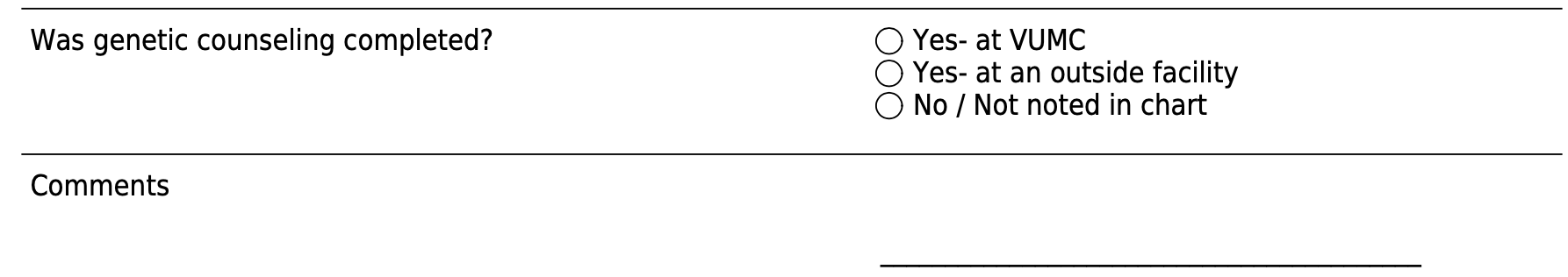


**
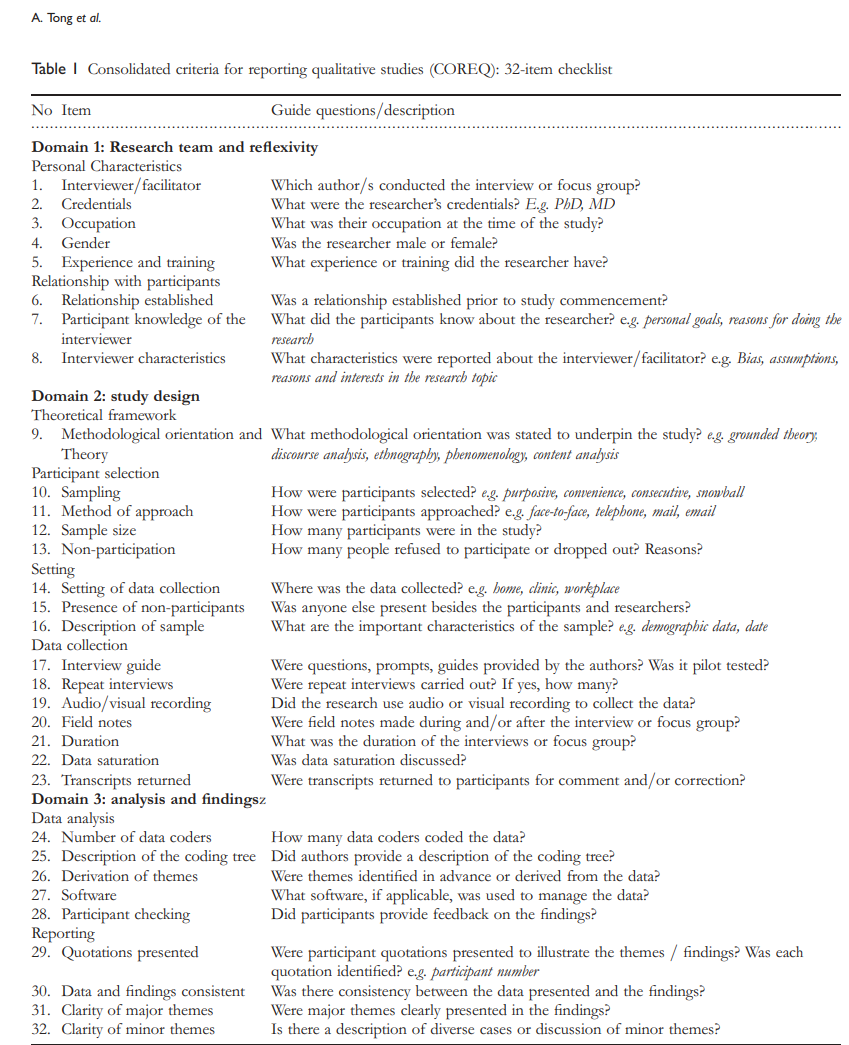
**
